# Proposed minimal standards for description of new taxa of the class Halobacteria

**DOI:** 10.1099/ijsem.0.006290

**Published:** 2024-03-08

**Authors:** Heng-Lin Cui, Jing Hou, Mohammad Ali Amoozegar, Mike L. Dyall-Smith, Rafael R. de la Haba, Hiroaki Minegishi, Rafael Montalvo-Rodriguez, Aharon Oren, Cristina Sanchez-Porro, Antonio Ventosa, Russell H. Vreeland

**Affiliations:** 1 School of Food and Biological Engineering, Jiangsu University, Zhenjiang 212013, PR China; 2 Department of Microbiology, School of Biology and Center of Excellence in Phylogeny of Living Organisms, College of Science, University of Tehran, Tehran 14178-64411, Iran; 3 Veterinary Biosciences, Melbourne Veterinary School, Faculty of Science, University of Melbourne, Parkville, 3010, Australia; 4 Department of Microbiology and Parasitology, Faculty of Pharmacy, University of Sevilla, Sevilla, Spain; 5 Department of Applied Chemistry, Faculty of Science and Engineering, Toyo University, Kawagoe, Japan; 6 Biology Department, Box 9000, University of Puerto Rico, Mayagüez, PR 00681, USA; 7 Department of Plant and Environmental Sciences, The Institute of Life Sciences, The Edmond J. Safra Campus, The Hebrew University of Jerusalem, Jerusalem 9190401, Israel; 8 Eastern Shore Microbes, 15397 Merry Cat Lane, Post Office Box 216, Belle Haven, VA 23306, USA

**Keywords:** *Archaea*, *Halobacteria*, phylogeny, taxonomy

## Abstract

Halophilic archaea of the class *Halobacteria* are the most salt-requiring prokaryotes within the domain *Archaea*. In 1997, minimal standards for the description of new taxa in the order *Halobacteriales* were proposed. From then on, the taxonomy of the class *Halobacteria* provides an excellent example of how changing concepts on prokaryote taxonomy and the development of new methods were implemented. The last decades have witnessed a rapid expansion of the number of described taxa within the class *Halobacteria* coinciding with the era of genome sequencing development. The current members of the International Committee on Systematics of Prokaryotes Subcommittee on the Taxonomy of *Halobacteria* propose these revisions to the recommended minimal standards and encourage the use of advanced technologies in the taxonomic description of members of the *Halobacteria*. Most previously required and some recommended minimal standards for the description of new taxa in the class *Halobacteria* were retained in the present revision, but changes have been proposed in line with the new methodologies. In addition to the 16S rRNA gene, the *rpoB′* gene is an important molecular marker for the identification of members of the *Halobacteria*. Phylogenomic analysis based on concatenated conserved, single-copy marker genes is required to infer the taxonomic status of new taxa. The overall genome relatedness indexes have proven to be determinative in the classification of the taxa within the class *Halobacteria*. Average nucleotide identity, digital DNA–DNA hybridization, and average amino acid identity values should be calculated for rigorous comparison among close relatives.

## Introduction

Halophilic archaea of the class *Halobacteria* are the most salt-requiring prokaryotes within the domain *Archaea*, and most of them flourish in hypersaline environments containing salt concentrations up to saturation [1,2]. They grow in a wide variety of hypersaline niches, including those with low nutrient availability, low oxygen solubility, high visible and ultraviolet radiation, acidic or alkaline pH, low and high temperatures, and those with heavy metals and other inhibitory compounds [1,3]. As of December 2023, the International Committee on Systematics of Prokaryotes (ICSP) Subcommittee on the Taxonomy of *Halobacteria* supports the taxonomic opinion that divides the class *Halobacteria* into two orders, nine families, 82 genera, and 357 species with validly published names (Table 1), being the largest class in the domain *Archaea* [1,3,6]. Most members of the *Halobacteria* are neutrophilic, while about 50 species are alkaliphilic, and only the representatives of the genus *Halarchaeum* are moderately acidophilic [7]. The great majority prefer to grow aerobically while only four prefer microaerophilic or strictly anaerobic conditions (*Halorhabdus tiamatea*, *Halanaeroarchaeum sulfurireducens*, *Halodesulfurarchaeum formicicum*, and * Natranaeroarchaeum sulfidigenes*) [8,11]. The colonies of nearly all members of the class are orange, red, or pink due to the presence of bacterioruberin carotenoids, but some species lack pigmentation [1,12, 13]. They exhibit diverse cell morphologies such as rod, coccus, disc, triangular, square, and pleomorphic shapes [1,14]. The recently described *Halocatena* and *Actinarchaeum* (name effectively published as *Actinoarchaeum* and corrected by the List Editors of the *International Journal of Systematic and Evolutionary Microbiology*) were surprisingly found to be filamentous and produced white spores from mycelia [15,16]. The aerobic and facultatively anaerobic representatives of the *Halobacteria* are chemoorganotrophic, using amino acids or carbohydrates as carbon sources, while the strictly anaerobic members are chemoorganoheterotrophic, utilizing acetate as electron donor (carbon source) and elemental sulfur as electron acceptor, or chemolithoheterotrophic, using formate or hydrogen as electron donors and elemental sulfur, thiosulfate, or dimethyl sulfoxide as electron acceptors [9,10]. These versatile morphologic and metabolic features expanded the previously recognized diversity within the class *Halobacteria*.

**Table 1.** Orders, families, and genera with validly published names, not including synonyms, included into the class *Halobacteria* as of December 2023, based on the classification proposed by Cui *et al.* [4]

Class	Order	Family	Genus, recommended three-letter abbreviation, and no. of species
*Halobacteria*	*Halobacteriales*	*Haladaptataceae*	*Haladaptatus* (*Hap.*) (6)*Halomicrococcus* (*Hmo.*) (1)*Halorussus* (*Hrs.*) (15)
		*Haloarculaceae*	*Halapricum* (*Hpr.*) (2)*Haloarcula* (*Har.*) (13)*Halocatena* (*Hct.*) (3)*Halococcoides* (*Hcs.*) (1)*Haloglomus* (*Hgl.*) (3)*Halomarina* (*Hmr.*) (3)*Halomicroarcula* (*Hma.*) (8)*Halomicrobium* (*Hmc.*) (4)*Halorarius* (*Hor.*) (2)*Halorhabdus* (*Hrd.*) (5)*Halorientalis* (*Hos*) (7)*Halosegnis* (*Hsg.*) (2)*Halosimplex* (*Hsx.*) (6)*Halovenus* (*Hvn.*) (4)*Natronomonas* (*Nmn.*) (8)*Salella* (*Sll.*) (1)*Salinibaculum (*Sbl.*) (1)*Salinirubellus* (*Srb.*) (1)*Salinirussus* (*Srs.*) (1)
		*Halobacteriaceae*	*Halanaeroarchaeum* (*Haa.*) (1)*Halarchaeum* (*Hla.*) (6)*Halobacterium* (*Hbt.*) (8)*Halocalculus* (*Hcl.*) (1)*Halodesulfurarchaeum* (*Hda.*) (1)*Salarchaeum* (*Sar.*) (1)
		*Halococcaceae*	*Halalkalicoccus* (*Hac.*) (1)*Halococcus* (*Hcc.*) (10)
		*Haloferacaceae*	*Halalkaliarchaeum* (*Hla.*) (1)*Halalkalirubrum* (*Hak.*) (1)*Halegenticoccus* (*Hgc.*) (2)*Halobaculum* (*Hbl.*) (7)*Halobellus* (*Hbs.*) (10)*Halobium* (*Hbm.*) (2)*Haloferax* (*Hfx.*) (13)*Halogeometricum* (*Hgm.*) (4)*Halogranum* (*Hgn.*) (4)*Halohasta* (*Hht.*) (3)*Halolamina* (*Hlm.*) (6)*Halonotius* (*Hns.*) (4)*Haloparvum* (*Hpv.*) (2)*Halopelagius* (*Hpl.*) (3)*Halopenitus* (*Hpt.*) (3)*Haloplanus* (*Hpn.*) (9)*Haloprofundus* (*Hpd.*) (5)*Haloquadratum* (*Hqr.*) (1)*Halorubrum* (*Hrr.*) (38)*Natronocalculus* (*Ncl.*) (1)*Salinigranum* (*Sgn.*) (4)*Salinirubrum* (*Srr.*) (1)
		*Halorubellaceae*	*Haloarchaeobius* (*Hab.*) (5)*Halorubellus* (*Hrb.*) (2)
		*Natronoarchaeaceae*	*Halostella* (*Hsl.*) (4)*Natranaeroarchaeum* (*Naa.*) (2)*Natronoarchaeum* (*Nac.*) (4)*Salinarchaeum* (*Saa.*) (2)
		*Natrialbaceae*	*Halobiforma* (*Hbf.*) (3)*Halopiger* (*Hpg.*) (5)*Halosolutus* (*Hss.*) (3)*Halostagnicola* (*Hst.*) (4)*Haloterrigena* (*Htg.*) (4)*Halovivax* (*Hvx.*) (5)*Natrarchaeobaculum* (*Nbl.*) (2)*Natrarchaeobius* (*Nar.*) (2)*Natrialba* (*Nab.*) (7)*Natribaculum* (*Nbl.*) (3)*Natrinema* (*Nnm.*) (22)*Natronobacterium* (*Nbt.*) (2)*Natronobeatus* (*Nbs.*) (1)*Natronobiforma* (*Nbf.*) (1)*Natronococcus* (*Ncc.*) (4)*Natronolimnobius* (*Nln.*) (1)*Natronolimnohabitans* (*Nlh.*) (1)*Natrononativus* (*Nnt.*) (1)*Natronorubrum* (*Nrr.*) (8)*Natronosalvus* (*Nsv.*) (5)*Salinadaptatus* (*Sad.*) (1)*Salinilacihabitans* (*Slc.*) (1)*Saliphagus* (*Spg.*) (1)
	*Halorutilales*	*Halorutilaceae*	*Halorutilus* (*Hrt.*) (1)

* The three-letter abbreviation *Sll.* is first proposed here.

The current minimal standards for the description of new taxa of halophilic archaea of the class *Halobacteria* were proposed in 1997 [17]. From then on, the taxonomy of the group provides an excellent example of how changing concepts on prokaryote taxonomy and the development of new methods are implemented [18]. Dozens of novel taxa were described by sampling from a wider range of geographically distinct sites and using improved cultivation methods to elucidate the diversity of halophilic archaeal communities in different saline environments around the world in the last decades. Meanwhile, the amount of whole genome data of members of the *Halobacteria* in databases is increasing quickly with the rapid development of low-cost prokaryotic genome sequencing. Comparative genomic and phylogenomic analyses can result in the revision of previously incorrect classifications of strains, species, and even higher taxa [19]. The current recommended minimal standards for the description of novel members of the class should be revised in line with the new methodologies used in the genomic era [20]. Therefore, the ICSP Subcommittee on the Taxonomy of *Halobacteria* proposes this updated minimal standards document for taxonomic description of new taxa of the class *Halobacteria*.

## General principles

One of the purposes of the Subcommittee is to recommend minimal standards for the description of new taxa [21]. This updated version of the minimal standards is based on polyphasic taxonomy, a consensus approach to the systematics of prokaryotes, and incorporated from the previous version of the proposed minimal standards [17]. To keep the continuity of haloarchaeal taxonomy and the comprehensive description of new taxa, the tests described as required in the earlier recommended minimal standards document were retained in this revision with the exception of antimicrobial susceptibility tests. Some previously recommended tests, such as electron microscopy, anaerobic growth in the presence of dimethyl sulfoxide (DMSO) or trimethylamine *N*-oxide (TMAO), and urease activity were also retained (Table 2). In the pre-genomic era, 16S rRNA gene sequence identities from 97 to 98.65 % were used as the most important thresholds for species demarcation [22]; however, intragenomic 16S rRNA gene sequence heterogeneity was found to be a rather common feature among the *Halobacteria* [23]. Phylogenetic analyses based on these heterogeneous 16S rRNA genes may lead to inaccurate phylogenies. In addition to the 16S rRNA gene, the RNA polymerase subunit B′ (*rpoB′*) gene, a single-copy conserved gene, was selected as an appropriate alternative phylogenetic marker for their identification [24]. Whole genome sequence data provide objective and reliable information for the taxonomy of prokaryotes [25]. Concatenated single-copy orthologous protein-coding genes present in the genome sequences of members of the *Halobacteria* were successfully used to infer the taxonomic ranks within the class *Halobacteria* [4,26, 27]. The overall genome relatedness indexes (OGRIs), such as average nucleotide identity (ANI), average amino acid identity (AAI), and digital DNA–DNA hybridization (dDDH), are currently widely used to delineate novel species [25,27]. These characteristics revealed by traditional and modern techniques or approaches should be fully considered when describing novel taxa.

**Table 2.** Proposed minimal standards for description of new taxa of the class *Halobacteria*

Category	Characteristic	Importance
Cultivation and isolation	Characteristic of collected sample	Required
	Cultivation method	Required
	Isolation strategy	Required
Morphology	Colonial morphology	Required
	Pigmentation	Required
	Cell morphology	Required
	Motility	Required
	Gram stain	Required
	Electron micrograph	Recommended
Growth conditions	Aerobic or anaerobic	Required
	NaCl concentration to prevent cell lysis	Required
	Salt concentration optimum and range for growth	Required
	pH optimum and range enabling growth	Required
	Temperature optimum and range enabling growth	Required
Nutrient requirements	Anaerobic growth with nitrate	Required
	Reduction of nitrate to nitrite	Required
	Formation of gas from nitrate	Required
	Anaerobic growth with arginine	Required
	Anaerobic growth with DMSO	Recommended
	Anaerobic growth with TMAO	Recommended
	Anaerobic growth with sulfur compounds (anaerobic strains only)	Required
	Utilization of carbohydrates as single carbon and energy sources	Required
	Utilization of amino acids as single carbon, nitrogen, and energy sources	Recommended
Biochemical activities	Catalase and oxidase activities	Required
	Acid production from carbohydrates	Required
	Indole formation	Required
	Hydrogen sulphide production	Required
	Starch hydrolysis	Required
	Gelatin hydrolysis	Required
	Casein hydrolysis	Required
	Tween 80 hydrolysis	Required
	Urease activity	Recommended
Polar lipid profiles	Phospholipid composition	Required
	Types of glycolipids	Required
Phylogeny	16S rRNA gene-based phylogeny	Required
	*rpoB′* gene-based phylogeny	Required
Genomics	Functional annotation and physiological prediction	Required
	G+C content	Required
	Phylogenomic analysis	Required
	Overall genome relatedness indexes (ANI, AAI, dDDH)	Required

## Cultivation and isolation

Halophilic archaea are widely distributed in natural or artificial hypersaline environments around the world [1,2]. Geographical and temporal information (latitude, longitude, altitude, and time of the year) and physico-chemical properties (pH, salinity/conductivity, and temperature) of habitats, and sampling and storage methods should be included in the descriptions. Culture medium, incubation conditions, and time required for growth should be described in detail along with the isolation strategy and purification techniques. The cultivation and isolation of two strains of *Haloquadratum walsbyi* was destined to become a classic in the taxonomy of the *Halobacteria* [28,29]. One strain was obtained by using an extinction–dilution cultivating method while the other was recovered by serial enrichment. Both strains could not grow well on agar plates while exhibiting the best growth in liquid low-nutrient media containing pyruvate [30]. Researchers are encouraged to isolate at least two related strains from different environments for the description of new taxa. For long-term preservation, the practice of suspending fresh cultures in a suitable liquid medium containing 15–20 % (w/v) glycerol and storage at −80 °C is very practical and easy to accomplish in regular laboratories. Preservation storage in liquid nitrogen or freeze-drying can be conducted according to the relevant instructions from public culture collections. The proposed type strains should be deposited in two or more public culture collections in different countries with no restrictions on availability.

## Morphology

The colonies of most members of the *Halobacteria*, especially the pigmentation with shades of red, are impressive compared with other archaea and bacteria. The morphological features of the colony, such as pigmentation, diameter, elevation, consistency, and opacity, should be described according to microbiological standards. The salt–milk agar medium containing skim milk facilitates the recognition of coloured colonies on the agar plate with a white background [31]. This method deserves to be more widely used for the characterization of those representatives that are able to grow on this medium. Cells of most species are easy to lyse or deform in distilled water or hypotonic solution. Gram staining should be conducted according to Dussault’s improved technique for staining halophilic archaea [32]. Fresh cultures in the exponential growth phase should be used to prepare the wet mounts to be examined by phase-contrast microscopy to observe cell shape, motility, and presence of gas vesicles. Special microscope slides coated with melted agarose are recommended for microscopic observation and photography of living cells. The protocol given in section 8.1.2 ‘Phase contrast light microscopic examination on agarose coated slides’ on pp. 126–127 of *The Halohandbook* [33] works well. Electron microscopy is recommended to examine members with unique cell shapes, such as species of *Haloquadratum* and *Halocatena* [15,30]. For scanning electron microscopic examination, a robust method is as follows: a 0.5 ml fresh culture is fixed overnight at 4 °C by adding glutaraldehyde to a final concentration of 5 %. The fixed sample (5 µl) should be smeared on a polylysine-coated coverslip and air-dried. The coverslips loaded with cells should be desalted by immersing in 2.5 % glutaraldehyde for 5 min, then serially dehydrated in 40, 70, 90, and 100 % ethanol solutions (10 min at each stage), critical-point dried, and viewed on a scanning electron microscope. To take transmission electron microscopy images, good results have been achieved using fresh cells stained with 0.5 % uranyl acetate in 25 % (w/v) NaCl for 30 s, then desalted with 2 % acetic acid, and examined under a transmission electron microscope.

## Growth conditions

Oxygen may be an important growth factor in the cultivation of members of the *Halobacteria*. The oxygen requirement of isolates can be distinguished easily by checking their growth under aerobic or anaerobic conditions. Sodium salt (NaCl) and magnesium salt (MgCl_2_ or MgSO_4_) are two major common salts required [34]. Most species require at least 8 % (w/v) NaCl concentration for growth, and most of them lyse in a lower salt concentration medium. NaCl solutions with serial concentrations from 1–15 % (w/v) should be used to determine the minimum salt concentration to prevent cell lysis. For measurement of the optimal concentration and range of NaCl enabling growth, a modified medium is recommended containing NaCl at concentrations from 5–35 % (at intervals of 2–3 %) supplemented with 0.1 g l^−1^ MgCl_2_ or MgSO_4_. The Mg^2+^ concentrations supporting growth and the optimal concentration should be tested in modified medium containing different concentrations of MgCl_2_ (0–1.0 M) at the optimal NaCl concentration. The pH range (pH 5.0–10.0, in increments of 0.5) and optimal value for growth should be tested in a modified medium supplemented with suitable buffers to maintain stable pH values. The optimum temperature and temperature range for growth should be observed by cultivating the organisms at 15–60 °C. If the growth characteristics of certain strains are found to be close to the minimum or the maximum, the testing ranges of the above factors should be enlarged.

## Nutrient requirements

Many aerobic members of the *Halobacteria* can grow anaerobically by using alternative electron acceptors such as nitrate, DMSO, or TMAO, or by fermenting l-arginine [17]. Some representatives utilize elemental sulfur, thiosulfate, or DMSO as electron acceptors [9,35]. Support of growth by the above electron acceptors should be determined according to the previously described methods [10,17].

Many members of the *Halobacteria* are chemoorganotrophic utilizing carbohydrates as single carbon and energy sources, or amino acids as single carbon, nitrogen, and energy sources. Substrates to be tested may include d-glucose, d-mannose, d-galactose, d-fructose, l-sorbose, d-ribose, d-xylose, maltose, sucrose, lactose, starch, glycerol, d-mannitol, d-sorbitol, acetate, pyruvate, dl-lactate, succinate, l-malate, fumarate, citrate, glycine, l-alanine, l-arginine, l-aspartate, l-glutamate, l-lysine, and l-ornithine. Yeast extract (0.1 g l^−1^ or less), KH_2_PO_4_ (0.05 g l^−1^), NH_4_Cl (0.5 g l^−1^, for neutrophiles), or NaNO_3_ (0.5 g l^−1^, for alkaliphiles) should be added to avoid depletion of growth factors, phosphorus, and nitrogen. A suitable buffer should be used in the determination of metabolized sugars, but buffers should be omitted in the testing of acid production from sugars and sugar alcohols [17]. Phenol red is a suitable pH indicator in such tests. Commercial miniaturized systems such as API (bioMérieux) or Biolog are not suitable for testing substrate utilization by representatives of the *Halobacteria* as they do not always perform reliably for high-salt media.

## Biochemical activities

Miscellaneous biochemical tests for catalase, oxidase, formation of indole, and hydrolysis of starch, gelatin, casein, and Tween 80 required previously are still routinely used in descriptions of new taxa. However, earlier recommended tests for phosphatase, urease, β-galactosidase, and lysine and ornithine decarboxylase activities were seldom conducted. Considering that diverse phosphatase, galactosidase, and decarboxylase activities can be functionally annotated by using the Rapid Annotation using Subsystem Technology (rast) server [36] based on genome information, these tests are not recommended in the revised standards except for urease, a characteristic enzyme in some species [37]. The traditional tests for catalase, oxidase, reduction of nitrate to nitrite, formation of gas from nitrate, indole formation, and hydrolysis of casein, gelatin, starch, and Tween 80 are maintained. The test for hydrogen sulfide production from thiosulfate that was not mandatory or recommended in the former minimal standards is now required in the description of new taxa [38,39]. Type strains of related species should be selected as positive and negative controls in the biochemical tests under the same experimental conditions simultaneously. As indicated above, commercial miniaturized systems, such as API (bioMérieux), are unadvisable for testing the biochemical activities of representatives of the class *Halobacteria*.

## Polar lipid profiles

The polar lipids of members of the *Halobacteria* class account for about 90 % of total lipids and consist of 2,3-diphytanyl-*sn*-glycerol analogues of phospholipids and glycolipids [40,42]. The phospholipids mainly consist of C_20_C_20_ (diphytanyl), C_20_C_25_ (phytanyl-sesterterpanyl), or C_25_C_25_ (disesterterpanyl) diether analogues of phosphatidic acid (PA), phosphatidylglycerol (PG), phosphatidylglycerol phosphate methyl ester (PGP-Me), and phosphatidylglycerol sulfate (PGS) [43,44]. PA, PG, and PGP-Me are present in all known taxa, while PGS is found in many, but not in all genera and species. Different glycolipids are found in different genera, including mannosyl glucosyl diether (DGD-1), another diglycosyl diether (DGD-2), sulfated mannosyl glucosyl diethers (S-DGD-1, S-DGD-3, S-DGD-5), disulfated mannosyl glucosyl diether (S_2_-DGD), galactosyl mannosyl glycosyl diether (TGD-l), glycosyl mannosyl glucosyl diether (TGD-2), sulfated galactosyl mannosyl glucosyl diether (S-TGD-1), and sulfated galactosyl mannosyl galactofuranosyl glucosyl diether (S-TeGD) [12,40,44]. Alkaliphilic species often lack glycolipids. Some species were found to contain cardiolipins such as bisphosphatidylglycerol (BPG) and phospholipid dimers composed of the glycolipid esterified to PA (S-DGD-PA, S-TGD-PA) [45,47]. These diverse polar lipids are the most important chemotaxonomic markers for the classification of the members of the class *Halobacteria*.

Considering the polar lipid composition may be affected by growth temperature, the tested strains should be cultivated under optimal growth conditions [48]. The polar lipids are recommended to be extracted using the Bligh and Dyer method [49] as modified for extreme halophiles [50], purified using acetone precipitation and analysed by using one-dimensional and two-dimensional thin-layer chromatography (TLC) [51]. The specific staining reagents to detect phospholipids and glycolipids should be freshly prepared [52,53]. Two-dimensional TLC can be used to show how many polar lipids are present in the strains under study, and one-dimensional TLC may enable the comparison of polar lipids present in the strains under study with those in the reference strains. The polar lipid components of new strains can also be identified and confirmed by using matrix-assisted laser desorption ionization–time of flight/mass spectrometry (MALDI-TOF/MS) [46,47].

## Phylogeny

As a highly conserved housekeeping gene, the 16S rRNA gene served in the past as the most important molecular marker for inferring archaeal phylogeny [54]. The 16S rRNA genes are still considered as the most important molecular indicators in the current identification of the members of the class. As intragenomic heterogeneity of 16S rRNA genes may be an ancient and stable trait in many lineages of the class *Halobacteria* [55], and to minimize the chance of any errors or unrecognized culture contamination, the diverse 16S rRNA genes of each undetermined strain should be retrieved from the genomes and also separately sequenced by the Sanger method and identified by comparison with those of already described species. The full-length sequences should be deposited in a public database (GenBank/EMBL/DDBJ), with the accession numbers included in the species description. The 16S rRNA gene sequence identity values between unidentified strains and related taxa should be calculated. A sequence identity of 98.65 % represents the threshold for species demarcation [22].

In contrast to the 16S rRNA gene, the *rpoB′* gene has not been detected in multiple copies in any genome of a member of the *Halobacteria* [56]. Therefore, the *rpoB′* gene may be a valuable alternative to the 16S rRNA gene in the identification. The full-length *rpoB′* gene sequence can be retrieved from the whole genome sequence or amplified by PCR using the primer pair HrpoB2-1420F/HrpoA-153R [24]. Sequence similarities equal to or lower than 86.2 % have been used for genera demarcation.

Phylogenetic trees inferred from the sequences of 16S rRNA and *rpoB′* genes should be reconstructed using the maximum-likelihood (ML) algorithm [57], although they must be double-checked using the neighbour-joining (NJ) and maximum-parsimony treeing methods [58,59]. The taxonomic status at the species and genus levels may be elucidated by inferring the 16S rRNA and *rpoB′* gene phylogenies. As the 16S rRNA and *rpoB′* genes have different perceived evolutionary rates, the tree topologies they generate may differ from each other, but in most cases, these differences do not affect the taxonomic assignment of the tested strains [24]. Instead, they can reveal different evolutionary lineages among related taxa. Due to the limited data information, 16S rRNA gene-based and *rpoB'* gene-based phylogenies cannot thoroughly elucidate the taxonomic status within the class *Halobacteria* at the family and order levels [60]. Genome-based phylogenetic analysis can make up for this deficiency [4].

## Genomics

As the most important source of taxonomic information, genome sequences allow a greater degree of accuracy in the classification of members of the *Halobacteria* [4,60]. The genomes of cultivated archaea and bacteria can easily be sequenced using next generation sequencing platforms in a short time and at low cost. Thus, the genome sequences of type strains are required for the description of new taxa by mainstream taxonomic journals. For the description of novel species, the whole genome sequence, either as a draft or as a complete genome, should be deposited in a public database. The quality of a genome used for taxonomy purposes should be up to the standards judged by genome size and sequencing depth of coverage [25]. The following minimal standards are recommended: presence of at least partial 16S rRNA gene sequence (the presence of the 23S, 16S, and 5S rRNA genes being highly recommended), agreement between 16S rRNA and *rpoB'* gene sequences extracted from the genome and the corresponding sequences determined by Sanger sequencing, >90 % completeness and <5 % contamination (using CheckM software), presence of at least 16 different tRNAs, contig no. <100, N50 >25 kb, and largest contig >100 kb.

The online rast server can be employed for functional annotation of the sequenced genomes [36]. The Kyoto Encyclopedia of Genes and Genomes (kegg) database may be used to identify the general metabolic pathways of the genomes [61]. The orthologous clusters (OCs) among isolated strains and their related species can be retrieved by using the online OrthoVenn3 [62]. Other state-of-the-art pangenome analysis tools such as the Enveomics collection [63], Anvi’o [64], get_homologues [65], and OrthoFinder [66] are also useful. Identification and characterization of other functional genes, such as polyhydroxyalkanoate (PHA) and carotenoid biosynthesis, is recommended by using BlastP (https://blast.ncbi.nlm.nih.gov/) based on high coverage and identity values. Some phenotypic characteristics related to physiology can be predicted by summarizing these annotated results. The DNA G+C content can be calculated from the nucleotide sequences of the whole genome, making the use of traditional techniques superfluous.

Phylogenomic analysis based on concatenation of conserved single-copy ubiquitous archaeal genes has established a standardized archaeal taxonomy and greatly improved our understanding of archaeal phylogeny [26]. The archaeal taxonomy of the Genome Taxonomy Database (GTDB) is recognized by more and more taxonomists. In a recent phylogenomic study, 30 single-copy orthologous proteins and 122 conserved single-copy marker proteins were selected from the genome sequences of type strains of the species of the class *Halobacteria* for reconstructing the OrthoFinder and GTDB trees, respectively [4]. This genome-based study elucidated the taxonomic relationship among current species with validly published names within the class *Halobacteria* at family and order levels. Additionally, robust phylogenomic analysis based on the concatenation of the translated sequences of the orthologous single-copy genes shared by the strains under study (core genes) has been successfully applied to clarify the taxonomic status of some genera of the class *Halobacteria* [27]. Any of these approaches involving the use of conserved and/or core single-copy molecular markers are recommended to be adopted for the phylogenomic analysis of isolated strains and their relatives within the class *Halobacteria*. The phylogenomic results can confirm or correct those based on 16S rRNA and *rpoB'* genes.

The current taxonomy has benefited from approaches based on comparative genomic analyses to calculate the evolutionary distances among species and to delineate prokaryotic taxa at family, genus, and species levels [27]. Comparison of the OGRI values among different taxa has widely been used in the classification of archaea and bacteria. The taxonomic problems that arose in the past in the genera *Natrinema* and *Haloterrigena* were clarified mainly by comparing the OGRI values among the current species of these two genera [27]. The dDDH, ANI, and AAI values among tested strains and the type strains of related taxa can be calculated using readily accessible online tools, such as the Genome-to-Genome Distance Calculator 3.0, the ANI calculator, and the AAI calculator, respectively [63,67]. The species boundary thresholds, 70 % of dDDH, 95–96 % of ANI, and 95 % of AAI, are recommended when proposing novel species. The corresponding AAI values, <45 %, 45–65 %, and 65–95 %, have been proposed for distinguishing family, genus, and species, respectively [68]. However, the proposed AAI values cannot be universally employed for the classification of all prokaryotes and should be adjusted accordingly for different taxa. For example, the cutoff value of ≤76 % AAI has been proposed for genus demarcation within the family *Natrialbaceae* [27].

## Use of three-letter abbreviations for names of genera

The ICSP Subcommittee on the Taxonomy of *Halobacteria* recommends the use of three-letter abbreviations of the names of the genera belonging to the class when the use of the same single-letter abbreviation for different genera in a single article may cause confusion. For lists of the recommended three-letter abbreviations see [5] and Table 1. Authors describing new genera should therefore propose an appropriate three-letter abbreviation that starts with the first letter of the name.

## Conclusions

Forty-six years ago, ‘*Halobacterium halobium*’ was found to belong to an ancient group of organisms no more related to bacteria than to eukaryotes by using comparative cataloguing of the 16S rRNA [69]. Nowadays, genome-based analyses can provide a deeper understanding of the evolution of the members of the class. Both the unity and the diversity of this special group of life forms should be comprehensively investigated by employing polyphasic approaches. To comply with Recommendation 30 of the International Code of Nomenclature of Prokaryotes (ICNP) [70], the ICSP Subcommittee on the Taxonomy of *Halobacteria* presents this revised proposed minimal standards for description of new taxa based on recent developments and advanced technologies. The aim of the updated minimal standards is to provide guidance on the description of new taxa belonging to the class *Halobacteria*, focusing on cultivation and isolation, morphology, growth conditions, nutrient requirements, biochemical activities, polar lipid profiles, phylogeny, and genomics, but without restricting freedom of taxonomic thought or action (Principle 1(4) of the ICNP).

## Abbreviations

AAI: average amino acid identity
ANI: average nucleotide identity
dDDH: digital DNA–DNA hybridization
ICNP: International Code of Nomenclature of Prokaryotes
ICSP: International Committee on Systematics of Prokaryotes
OGRI: overall genome relatedness index
